# FERMT1 promotes gastric cancer progression by activating the NF-κB pathway and predicts poor prognosis

**DOI:** 10.1080/15384047.2020.1792218

**Published:** 2020-07-29

**Authors:** Hua Fan, Shengjun Zhang, Yu Zhang, Wu liang, Bo Cao

**Affiliations:** a Department of Gastrointestinal Surgery, Affiliated Hospital of Yan’an University, Yan’an, Shaanxi, China; b Department of Medicine, Xi’an Jiaotong University, Xi’an, Shaanxi, China; c Basic College of Tongji Medical College, Huazhong University of Science and Technology, Wuhan, Hubei, China

**Keywords:** FERMT1, gastric cancer, NF-ΚB, poor prognosis, epithelial-mesenchymal transition

## Abstract

Recent studies have reported that FERMT1, a newly discovered adhesion protein, contributes to an aggressive phenotype in several solid malignancies. However, the function and regulatory mechanism of FERMT1 in gastric cancer remain unknown. We found that FERMT1 was overexpressed in gastric cancer tissues compared with normal tissues. Clinical data analysis indicated that the expression of FERMT1 correlated with the overall survival of gastric cancer patients. Patients with higher FERMT1 expression had lower survival rates than patients with lower FERMT1 expression. We established stable cell lines with FERMT1 knockdown and overexpression. In vitro and in vivo experiments indicated that knockdown of FERMT1 inhibited the proliferation, invasion, metastasis, and epithelial-mesenchymal transition of gastric cancer cells. Mechanistically, FERMT1 was found to activate NF-κB signaling by promoting the degradation of IκBα, thereby promoting gastric cancer. These results provide new evidence of the oncogenic effects of FERMT1 in gastric cancer and suggest that FERMT1 might be a promising target for gastric cancer treatment.

## Introduction

Gastric cancer (GC) has high malignancy and poor prognosis, and is the third most commonly diagnosed cancer worldwide.^1,2^ Patients with advanced GC have poor prognosis, with a 5-year survival rate of 20%.^3^ Despite substantial advances in early diagnosis and treatment in recent decades, GC remains one of the most threatening malignancies.^4^ Therefore, exploring the mechanisms of GC cell proliferation, invasion, and metastasis is important for the early diagnosis and treatment of GC.

FERMT1 is a newly discovered adhesion protein belonging to the kindlin family,^5–7^ which consists of Ezrin, Radisin, moesin and a pleckstrin homology domain, and a pleckstrin homology domain. FERMT1 is a mutant gene associated with Kindler syndrome.^8–12^ Although FERMT-1 was thought to be mainly expressed and distributed in epithelial cells,^13^ studies increasingly show that FERMT1 is also expressed throughout the digestive system. Moreover, increasing evidence indicates that the FERMT1 gene is involved in tumor proliferation, apoptosis, metastasis, and tumor angiogenesis, and it is considered to be an oncogene.^14^ FERMT1 is strikingly upregulated in colon cancer, in which it drives epithelial-mesenchymal transition (EMT) by activating the transcriptional activity of β-catenin.^15,16^ Sin et al. have suggested that increased expression of FERMT1 promotes tumor growth and invasion.^17^ In skin cancer, loss of FERMT1 expression facilitates tumor formation through inducing αvβ6-integrin-mediated TGFβ release and Wnt ligand expression.^18^ Recent studies have shown that FERMT1 is also expressed in many tumors, such as pancreatic cancer, lung cancer, and bladder cancer.^19^ However, FERMT1 has not been reported in GC. Therefore, we explored the role of FERMT1 in GC development to facilitate early clinical diagnosis and prognostic evaluation.

## Materials and methods

### Cell culture

The cell lines (BGC823, AGS, HGC27, SGC7901, MKN-45, and SGC-7901) were obtained from the Chinese Academy of Sciences (Shanghai, China). The cells were subcultured for less than half a year after the start of culture in our laboratory. All cell lines were maintained in a 37°C 5% CO_2_ incubator. RPMI-1640 medium (HyClone, USA) was mixed with 1% penicillin-streptomycin (Solarbio, China) and 10% fetal bovine serum (Gibco, USA) to culture the cells. The treatment with TNF-α (10 ng/ml, Sigma-Aldrich, USA) was performed for 24 h. Bay 11–7082 (10 μM, Abcam, Cambridge, UK) treatment was performed for 12 h.

### Clinical data

Data for a total of 42 patients with GC were collected from the Department of Gastrointestinal Surgery of Union Hospital, Tongji Medical College, Huazhong University of Science and Technology, after the patients signed a written informed consent form. Ethical approval was provided by the Ethics Committee of Tongji Medical College, Huazhong University of Science and Technology (Wuhan, China).

### Plasmid construction and transfection

FERMT1 shRNA and negative control were purchased from RiboBio (Guangzhou, China) and transfected into cells at 30% conﬂuence. The transfection reagent was Lipofectamine 2000 reagent (Invitrogen, USA). Western blotting and qRT–PCR were applied to confirm the knockdown efficiency of FERMT1. An expression vector encoding human FERMT1 and a control vector were obtained from Genechem, China.

### IHC

The immunohistochemistry (IHC) methods were as described elsewhere.^20^

### Western blot assay

The western blot assay procedure was consistent with previously described methods.^21,22^ Primary antibodies against FERMT1, *p*-P65, P65, E-cadherin, N-cadherin, vimentin, MMP9, MMP2, PCNA, IκBα, *p*-ERK1/2, and Cyclin D1 were purchased from BD Biosciences (San Jose, CA, USA), and the loading controls (H3 and GAPDH) were purchased from Proteintech (Rosemont, IL, USA).

### Real-time PCR

TRIzol reagent (Invitrogen, Carlsbad, CA, USA) was used for total RNA isolation. The cDNA was prepared with a PrimeScriptTM RT reagent kit (Takara, Dalian, China). GAPDH served as the reference. The method of real-time PCR was as described elsewhere.^23^ The primer sequences are shown in Supplementary Table 1.

### Transwell migration and invasion assays

The Transwell assay procedure was as previously described.^24,25^ This assay is commonly used to test the migratory and invasive abilities of cells. Matrigel (BD Biosciences, Sparks, USA) was used to precoat the upper surface of the Transwell chamber for the Transwell invasion assay. The number of cells that had migrated or invaded was calculated by viewing cells under a microscope in five random fields. All experiments were repeated in triplicate.

### Wound-healing assay

First, five uniform horizontal lines were marked behind the six-well plate. GC cells, in a quantity to ensure confluency overnight, were then added to the wells. The next day, scratches were made with a 200 μl sterile pipette tip perpendicular to the horizontal line on the back of the plate. The cells were washed, and serum-free medium was added. The plate was placed in a 37°C 5% CO_2_ incubator. Samples were collected at 0 h and 48 h, and pictures were taken.

### Cell viability assay

Cell Counting Kit-8 (CCK-8; Dojindo, Kumamoto, Japan) was used according to the instruction manual. Treated 3,000 cell suspension was added to a 96-well plate and cultured in an incubator for 1, 2, 3, 4, or 5 days. Then 10 μl CCK8 solution was added to each well and incubated for 1 h. The absorbance at 450 nm was detected with a microplate reader. All experiments were repeated in triplicate.

### Colony formation assay

A total of 500 treated cells were uniformly inoculated into a six-well plate and cultured for 10 days. After the cells were fixed with 1 ml of 4% paraformaldehyde for 20 minutes, an appropriate amount of crystal violet was added for 15 minutes, and then the staining solution was slowly washed with running water, and the cells were air-dried. Colonies were counted under an optical microscope. All experiments were repeated in triplicate.

### Xenograft assay

For the xenograft model, transfected GC cells were subcutaneously implanted into BALB/c nude mice (females at four weeks old; HFK Bio-Technology Co., Ltd, Beijing, China). At 21 days after implantation, tumors were removed and weighed after mice were sacrificed. For the metastasis model, transfected GC cells were injected into the mice via the tail vein. The mice were sacrificed after four weeks, and the lungs were removed for H&E staining.

### Statistical analysis

The data were analyzed in SPSS V23 (IBM, Armonk, NY, USA). Data are expressed as the mean ± standard deviation. Student’s *t*-test was applied to measure between-group differences. The correlation between FERMT1 and NF-κB was analyzed with Pearson correlation. A *P*-value ≤0.5 was considered statistically significant.

## Results

### FERMT1 is aberrantly expressed in GC tissues

**Figure 1.**
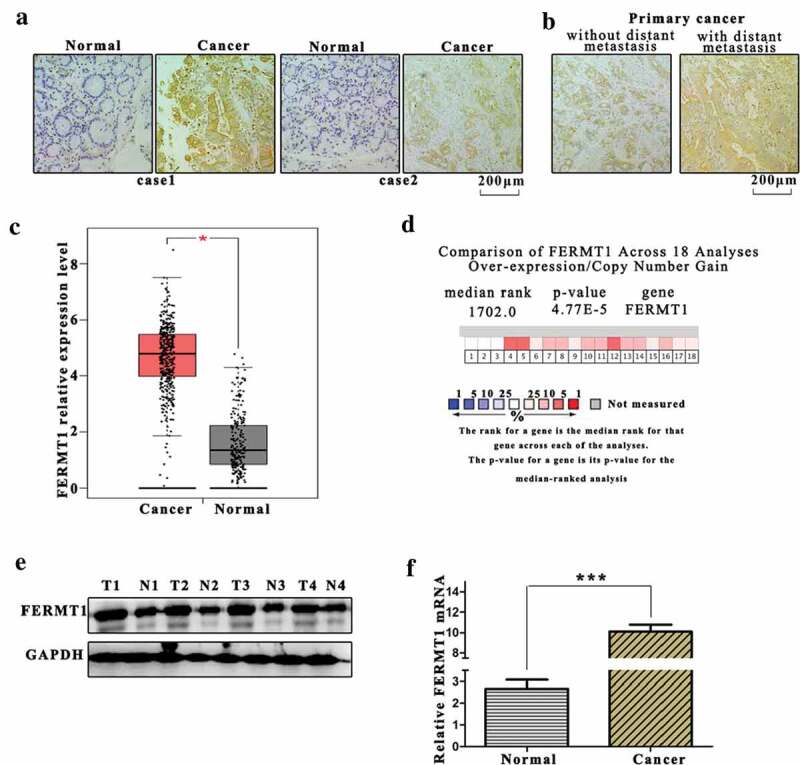
FERMT1 was overexpressed in gastric cancer tissues.

We examined 42 GC tissues and paired normal tissues by IHC to test the FERMT1 expression in GC. FERMT1 was notably elevated in GC tissues compared with normal tissues (Figure 1a). Overexpression of FERMT1 was observed in primary cancer with distant metastasis compared with primary cancer without distant metastasis (Figure 1b). We further detected the FERMT1 mRNA levels in 42 specimens by qRT-PCR and found that the mRNA level of FERMT1 was significantly upregulated in GC tissues compared with normal tissues (Figure 1c). Meanwhile, 18 analyzes from the Oncomine microarray database also indicated that FERMT1 was highly expressed in GC (Figure 1d). From western blotting and qRT-PCR experiments, we found that the FERMT1 protein and mRNA levels were significantly elevated in GC (Figure 1e,f). Together, these results illustrate that FERMT1 is overexpressed in patients with GC.

(a) IHC Images showed FERMT1 expression in adjacent normal gastric tissues and GC tissues of two patients. Scale bar: 200 μm. (b) IHC Images showed FERMT1 expression in primary GC tumors with or without distant metastasis. Scale bar: 200 μm. (c) The FERMT1 expression was analyzed in GC tissues and adjacent normal gastric tissues. (d) FERMT1 expression was showed by Meta-analysis in 18 analyses from four independent studies. Colored squares represented the median rank for FERMT1 across 18 analyses. (e) The FERMT1 protein level was detected by western blot in GC tissues and adjacent normal gastric tissues of 4 patients. (f) The FERMT1 mRNA level was detected by qRT-PCR in GC tissues and adjacent normal gastric tissues.

### Upregulation of FERMT1 is closely associated with the prognosis of patients with GC

**Figure 2.**
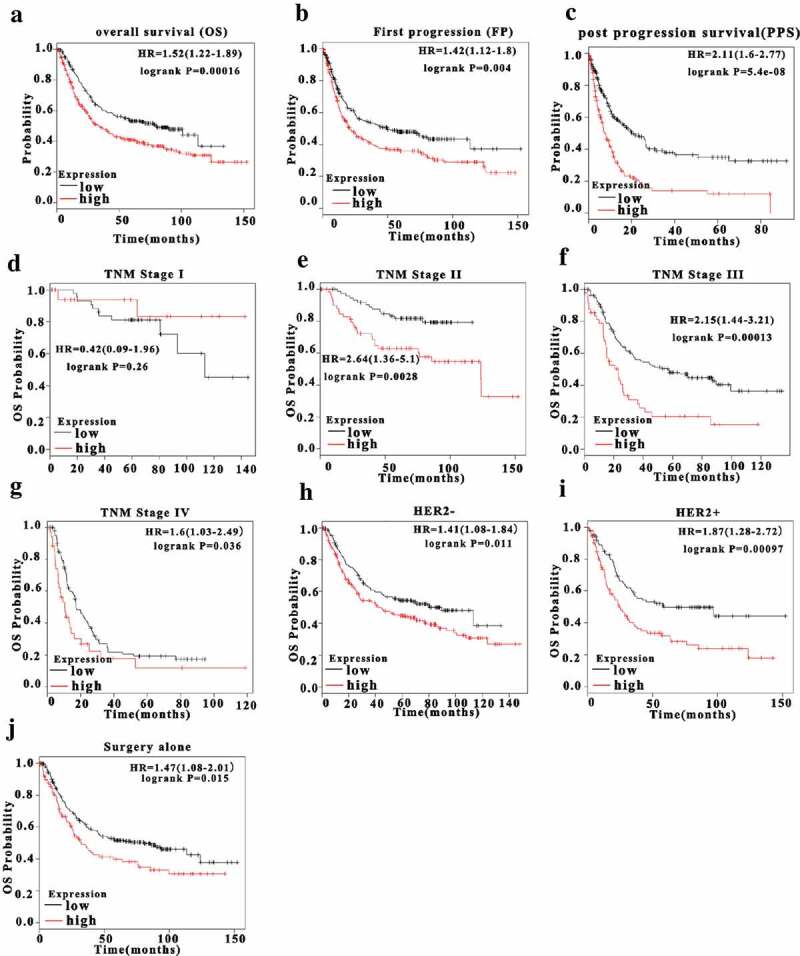
Upregulation FERMT1 is closely associated with poor prognosis of patients with GC.

Because FERMT1 showed aberrant expression in GC tissues, we wondered whether it might serve as an important prognostic marker in patients with GC. Consequently, we further investigated the link between FERMT1 expression and clinicopathological characteristics in GC tissues from 42 cases. The analyzes in Supplementary Table 1 indicated that overexpression of FERMT1 was correlated with T stage and N stage. Moreover, Kaplan–Meier analysis of data from the TCGA database indicated that patients with GC with higher FERMT1 mRNA levels have lower overall survival (OS) (Figure 2a), FP (Figure 2b) and PPS (Figure 2c) than those with lower levels of FERMT1. We next investigated the value of FERMT1 in predicting aggressive GC. High expression of FERMT1 was not associated with poorer OS in patients with early-stage (TNM I) GC (Figure 2d), possibly because of the limited number of patients included in the analysis. A close relationship was observed between upregulation FERMT1 and shorter OS in patients with TNM stage II–IV GC (Figure 2d-g). In survival analysis, similar trend was also detected for FERMT1 mRNA levels in HER2 negative and positive GC (Figure 2h,i). In addition, high expression of FERMT1 is correlated with low surgery OS (Figure 2j). Therefore, the high expression of FERMT1 is correlated with the OS rate in patients with GC.

(a) Kaplan–Meier survival analysis from the TCGA databases showed that FERMT1 expression had a correlation with overall survival in GC patients. (b) Kaplan-Meier analysis from the TCGA databases indicated that GC patients with high FERMT1 expression had a lower FP OS than those with low FERMT1 expression in GC patients. (c) Kaplan-Meier analysis from the TCGA databases indicated that GC patients with high FERMT1 expression had a notably low PP survival rate than those with low FERMT1 expression in GC patients. (d) Kaplan-Meier analysis from the TCGA databases showed that high expression of FERMT1 was not correlated with worse OS in GC patients with early-stage (TNM I). (e-g) Kaplan-Meier analysis from the TCGA databases showed that high expression of FERMT1 was related to worse OS in GC patients with TNM II (*P* = .0028) stage, TNM III (*P* = .00013) stage and TNM IV (*P* = .036) stage. (h-i) Kaplan-Meier analysis from the TCGA databases showed that high expression of FERMT1 was related to in GC patients with HER2- (*P* = .011) and HER2+ (*P* = .00097). (j) Kaplan-Meier analysis from the TCGA databases showed that high expression of FERMT1 was related to worse OS in GC patients with surgery (*P* = .015).

### FERMT1 promotes the proliferation and metastasis of GC cells

**Figure 3.**
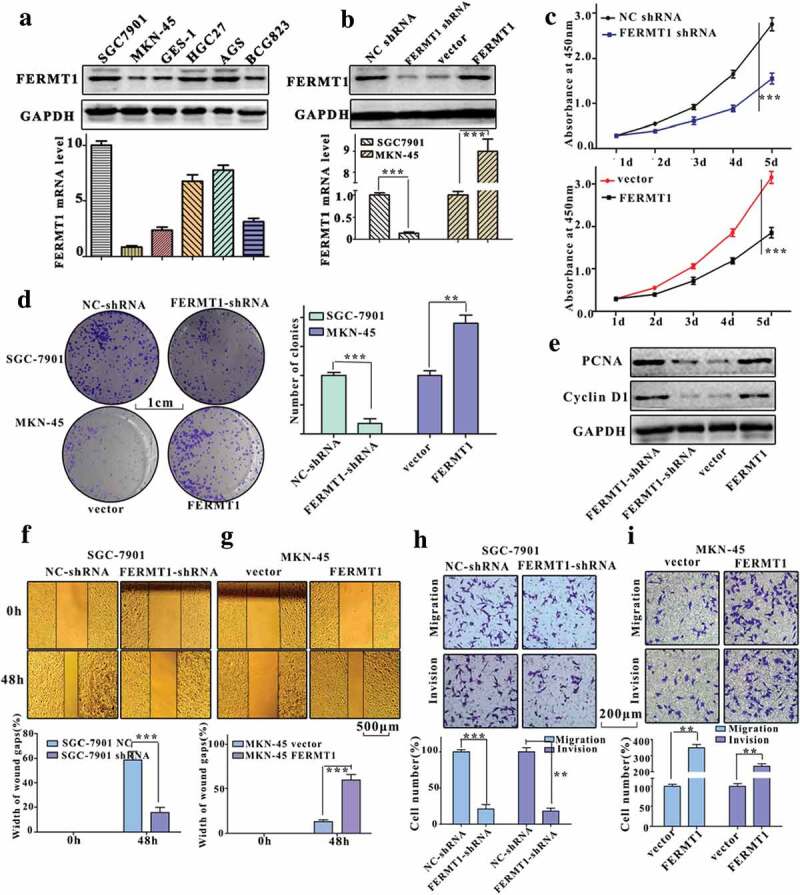
FERMT1 promotes the proliferation and metastasis of GC cells.

We next attempted to verify the effects of FERMT1 on the proliferation and invasion ability of GC cells. Using western blotting, we detected FERMT1 expression in six cell lines (SGC-7901, HGC27, AGS, SGC7901, MKN45, and BGC823) (Figure 3a). The FERMT1 protein level was much higher in the normal gastric epithelium cell line (GES1) than in other GC cells. We additionally found that FERMT1 showed the lowest protein and mRNA levels in MKN45 cells. Thus, SGC-7901 cells and MKN45 cells were chosen for FERMT1 knockdown and overexpression experiments. To knock down endogenous FERMT1 expression, we transfected SGC-7901 cells with shRNA. A plasmid encoding FERMT1 was also transfected into MKN45 cells to induce ectopic overexpression of FERMT1 (Figure 3b). CCK-8 and colony formation assays showed that the proliferative capability was significantly decreased in FERMT1-knockdown SGC-7901 cells but was notably increased in FERMT1-overexpressing MKN45 cells (Figure 3c,d). Proliferation-associated proteins (PCNA and Cyclin D1) were diminished in FERMT1 knockdown in SGC-7901 cells and elevated in FERMT1 overexpressing MKN45 cells (Figure 3e). In addition, reduced invasion and migration ability was found in FERMT1-knockdown SGC-7901 cells (Figure 3f-h), and increased invasion and migration capability was detected in FERMT1 overexpressing MKN45 cells by wound healing and Transwell analyzes (Figure 3g-i). Therefore, FERMT1 plays a vital role in the oncogenic activity of GC cells.

(a) The mRNA and protein level of FERMT1 were detected in six GC cell lines (SGC-7901, HGC27, AGS, SGC7901, MKN45 and BGC823) by qRT-PCR and western blot. (b) qRT-PCR and western blot were used to confirm the mRNA and protein expression of FERMT1 in SGC-7901 cells and MKN45 cells. (c-d) The proliferation of SGC-7901 cells and MKN45 cells was measured via CCK-8 and colony formation assay. (e) The protein level of PCNA and Cyclin D1 was detected in SGC-7901 cells and MKN45 cells by western blot. (f-g) The migration of SGC-7901 cells and MKN45 cells was measured via wound healing assay. (h-i) The migration and invasion of SGC-7901 cells and MKN45 cells were measured via transwell assay.

### FERMT1 promotes proliferation and metastasis of GC cells in vivo

**Figure 4.**
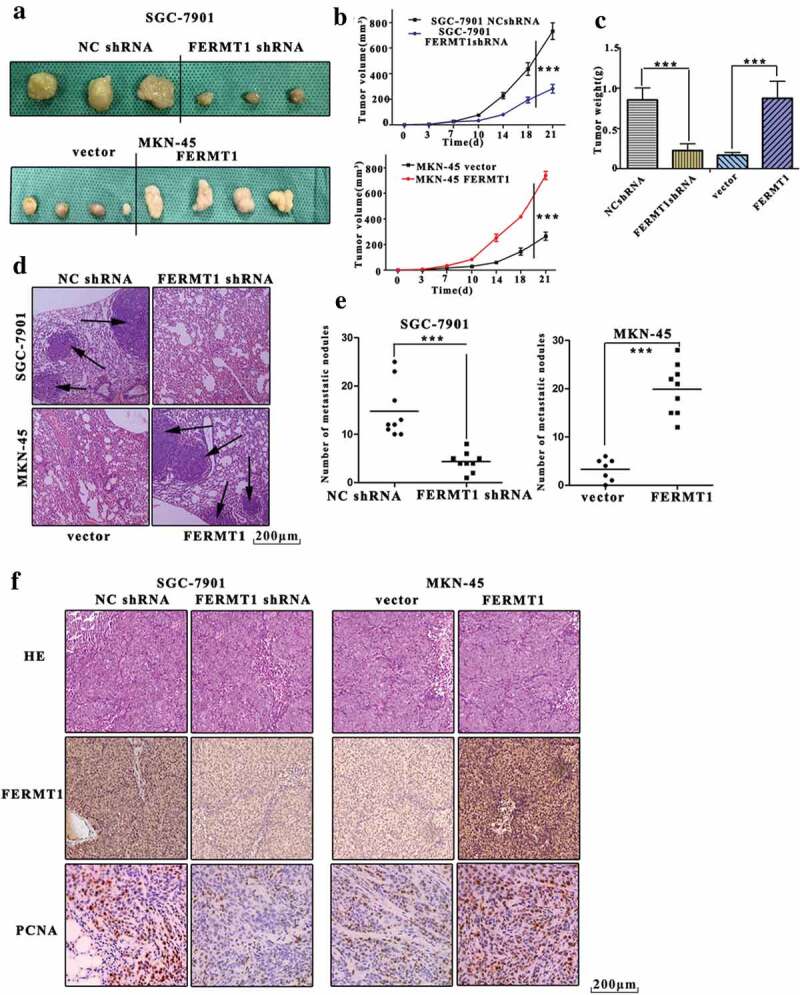
FERMT1 promotes proliferation and metastasis of GC cells in vivo.

We next evaluated the biological function of FERMT1 by accessing a xenograft model in nude mice. In Figure 4a, the tumors from SGC-7901-shFERMT1 cells showed reduced size, whereas increased tumor size was observed in FERMT1 overexpressing MKN45 cells. Mice implanted with SGC-7901-shFERMT1 cells, compared with controls, displayed lower tumor volume and weight. After overexpression of FERMT1, mice implanted with FERMT1 overexpressing MKN45 cells, compared with vector control cells, showed greater tumor volume and weight (Figure 4b,c). In addition, FERMT1-knockdown SGC-7901 cells and FERMT1 overexpressing MKN45 cells were injected via the tail vein into nude mice to assess the role of FERMT1 metastasis. The mice transfected with SGC-7901-shFERMT1 cells showed fewer lung metastatic nodules than the control mice (Figure 4d,e). Furthermore, the number of lung metastases in the FERMT1 overexpression group was lower than that in the control group. Through IHC experiments, we found that the expression of PCNA was also low in the FERMT1-low group and high in the FERMT1-overexpression group (Figure 4f). Together, the results above indicated that FERMT1 is a regulator of proliferation and metastasis in GC.

(a) 2 × 10^6^ stable SGC-7901 cells and MKN45 cells were injected subcutaneously into the nude mice. Scale bars, 1 cm. (b) The subcutaneous tumor volume was measured every 3 days. (c) The subcutaneous tumor weight was measured after mice were sacrificed. (d) 1 × 10^6^ stable SGC-7901 cells and MKN45 cells were injected into the nude mice via tail vein. The H&E images represented the metastatic foci in lungs. Scale bars, 200 μm. (e) The mRNA level of FERMT1 was detected in the subcutaneous tumor form NC shRNA, FERMT1 shRNA, Vector and FERMT1 group. (f) The H&E images represented the expression of FERMT1 and PCNA. Scale bars, 200 μm.

### FERMT1 activates the NF-κB pathway

**Figure 5.**
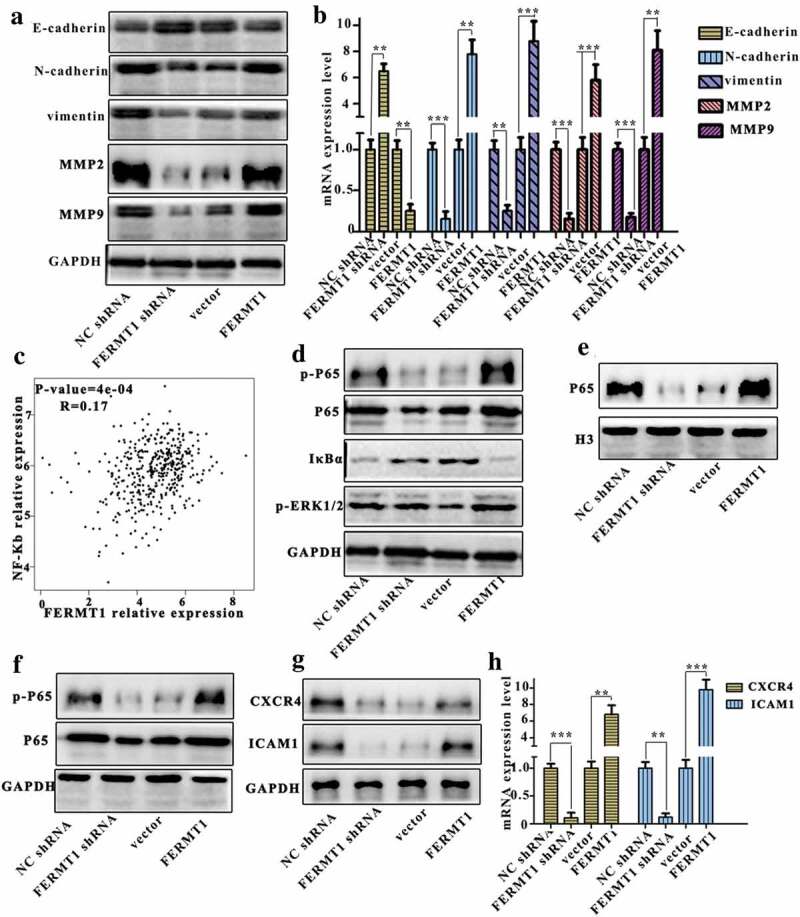
FERMT1 activates the NF-κB pathway through promoting the degradation of IκBα.

EMT is a vital driver of cancer development and distant metastasis.^26^ Therefore, we sought to determine whether FERMT1 might promote the proliferation and metastasis of GC cells through the regulation of EMT. The protein and mRNA levels of five representative EMT-related markers (E-cadherin, N-cadherin, Vimentin, MMP2, and MMP9) were detected in FERMT1 knockdown/control SGC-7901cells and FERMT1 overexpressing/control MKN45 cells. The E-cadherin expression was significantly increased in FERMT1-knockdown cells, whereas expression of N-cadherin, Vimentin, MMP2, and MMP9 was markedly decreased (Figure 5a,b). Meanwhile, the opposite result was observed in FERMT1-overexpressing cells: the E-cadherin expression was decreased, and the expression of N-cadherin, Vimentin, MMP2, and MMP9 was increased. These observations indicated that FERMT1 promotes EMT in GC cells.

(a) The protein level of E-cadherin, N-cadherin, vimentin, MMP9 and MMP2 was detected in SGC-7901 cells and MKN45 cells by western blot. (b) The mRNA level of E-cadherin, N-cadherin, vimentin, MMP9 and MMP2 was measured in SGC-7901 cells and MKN45 cells by qRT-PCR. (c) Pearson correlativity from the online database GEPIA a positive correlation between expression of FERMT1 and NF-κB. (d) The protein level of *p*-P65, P65, IκBα and *p*-ERK1/2 was detected in SGC-7901 cells and MKN45 cells by western blot. (e) The protein level of P65 was detected in SGC-7901 cells and MKN45 cells by western blot. (f) The protein level of *p*-P65 and P65 was detected in SGC-7901 cells and MKN45 cells by western blot. (g-h) The protein and mRNA level of CXCR4 and ICAM1 was detected in SGC-7901 cells and MKN45 cells by western blot and qRT-PCR.

Next, we sought to investigate the mechanism through which FERMT1 promotes EMT. FERMT1 has been reported to facilitate EMT by inducing the TGF signaling pathway in breast cancer, and NF-κB is an important signaling pathway through which TGF-β maintains the interstitial phenotype of tumor cells.^27,28^ Thus, we hypothesized that FERMT1 might promote EMT by inducing the NF-κB signaling pathway. The data from the online database Gene Expression Profiling Interactive Analysis (GEPIA) indicated that the expression of FERMT1 and NF-κB was positively correlated in GC (Figure 5c). In addition, the expression of p65 and *p*-p65 was significantly decreased in FERMT1 knockdown SGC-7901 cells, but increased in FERMT1 overexpressing MKN45 cells. We also observed that IκBα expression was notably upregulated after FERMT1 knockdown and downregulated after FERMT1 overexpression in GC cells (Figure 5d). No significant changes were observed in the expression of phosphorylated ERK1/2 in FERMT1 knockdown and FERMT1 overexpressing GC cells. To further verify the function of FERMT1 in the NF-κB signaling pathway, we detected the protein level of FERMT1 in the nucleus. Western blotting analysis indicated that downregulation of FERMT1 markedly disrupted the nuclear accumulation of p65, whereas upregulation FERMT1 notably increased the nuclear accumulation of p65 (Figure 5e). Similar results were found in xenograft tumors from mock knockdown and FERMT1 overexpressing GC cells: the protein levels of p65 and *p*-p65 were decreased in xenograft tumors formed from FERMT1 knockdown GC cells but were increased in xenograft tumors formed from FERMT1 overexpressing GC cells (Figure 5f), thus indicating that FERMT1 plays an important role in the activation of NF-κB signaling in vitro and in vivo. In addition, we found that the mRNA and protein levels of CXCR4 and ICAM1, two downstream targets of NF-κB,^10^ were significantly decreased in FERMT1 knockdown and increased in FERMT1 overexpressing GC cells (Figure 5g,h). Thus, FERMT1 is a crucial regulator of NF-κB.

### FERMT1 is an essential upstream regulator of the NF-κB signaling pathway in modulating GC development

**Figure 6.**
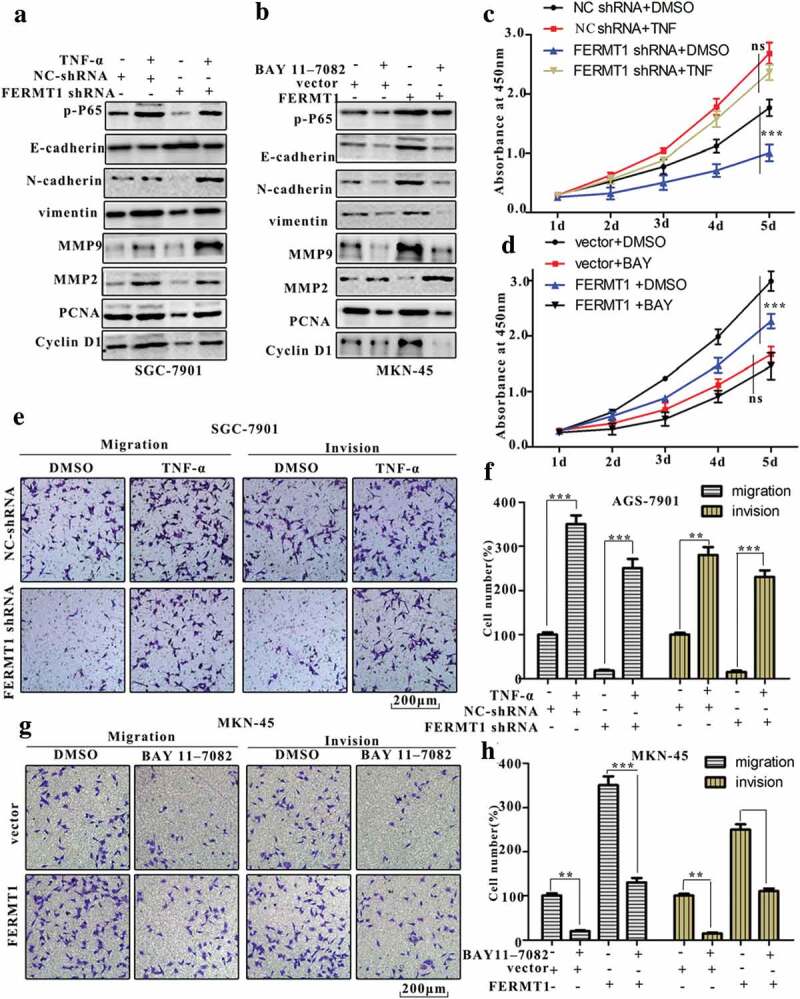
FERMT1 acts as a vital upstream regulator of the NF-κB signaling pathway to modulate the process of GC.

Next, we used activators and blockers of NF-κB signaling to explore the effect of FERMT1 on the NF-κB signaling pathway. Tumor necrosis factor-α (TNF-α), an activator of the NF-κB signaling pathway, was used to induce NF-κB. The expression of *p*-P65, N-cadherin, vimentin, MMP9, MMP2, PCNA, and Cyclin D1 significantly increased, whereas E-cadherin expression markedly decreased, after treatment with TNF-α. However, FERMT1 knockdown reversed the TNF-α induced expression of *p*-P65, N-cadherin, vimentin, MMP9, MMP2, PCNA, and Cyclin D1, and rescued the TNF-α- inhibited expression of E-cadherin in SGC-7901 cells (Figure 6a). A specific NF-κB inhibitor, BAY 11–7082, was used to repress NF-κB activity. As shown in Figure 6b, the treatment with BAY 11–7082 significantly downregulated *p*-P65, N-cadherin, vimentin, MMP9, MMP2, PCNA, and Cyclin D1, but upregulated E-cadherin. Furthermore, FERMT1 overexpression rescued the BAY 11–7082-inhibited expression of *p*-P65, N-cadherin, vimentin, MMP9, MMP2, PCNA, and Cyclin D1, as well as the BAY 11–7082-induced expression of E-cadherin in MKN-45 cells. Moreover, the proliferation, migration, and invasion ability of GC cells notably increased under treatment with TNF-α. FERMT1 knockdown reversed the increase in proliferation, migration, and invasion ability induced by TNF-α (Figure 6c-e). In addition, BAY 11–7082 markedly impaired the proliferation, migration, and invasion ability of GC cells. However, FERMT1 overexpression rescued the decrease of proliferation, migration and invasion ability that inhibited by BAY 11–7082 (Figure 6d-f). Therefore, these results indicated that FERMT1 facilitates the proliferation, migration, and invasion of GC cells by promoting the degradation of IκBα, thereby activating the NF-κB signaling pathway.

(a-b) The protein level of *p*-P65, E-cadherin, N-cadherin, vimentin, MMP9, MMP2, PCNA and Cyclin D1 were detected by western blot in SGC-7901 cells and MKN45 cells with or without TNF-α/BAY 11–7082 treatment. (c-d) The proliferation of SGC-7901 cells and MKN45 cells was measured by CCK8 assay with or without TNF-α/BAY 11–7082 treatment. (e-h) The migration and invasion of SGC-7901 cells and MKN45 cells were measured by transwell assay with or without TNF-α/BAY 11–7082 treatment.

## Discussion

The function and mechanisms of the newly discovered protein molecule FERMT1 have rarely been reported in GC. This study reveals a new mechanism for the role of FERMT1 in GC. Our study demonstrated that FERMT1 activates the NF-κB signaling pathway by promoting the degradation of IκBα, thus resulting in GC tumor progression.

We previously found that mutations in the FERMT1 gene (also known as KINDLIN-1) are the main cause of KS, a new type of bullous skin disease originally proposed by Theresa Kindler.^15,29,30^ FERMT1 has also been reported in various cancers. FERMT1 is upregulated in breast cancer, colon cancer, hepatocellular carcinoma, lung cancer, and pancreatic cancer.^31^ FERMT1 also appears to be a tumor driver in cutaneous epithelial cells. Tumor proliferation, invasion, and metastasis are important features of malignant tumors, and the processes and mechanisms are complex. Sin et al. have suggested that FERMT1 promotes lung metastasis and EMT progression through activating TGFβ signaling. In these cancers, TGFβ signaling appears to be a major factor in the carcinogenic effects of FERMT1. However, little is known about the mechanism through which FERMT1 exerts its oncogenic effect in GC. We established GC cell models with stable knockdown or overexpression of FERMT1. Our experiments indicated that FERMT1 promotes the proliferation, invasion, and metastasis of GC cells.

The EMT plays a crucial role in tumor metastasis and is a complex multi-path development process. FERMT1 promotes EMT in colon cancer.^15^ Similar results have been observed in GC cells. In our study, we also found that the expression of FERMT1 is associated with EMT markers, thus providing evidence that FERMT1 promotes EMT in GC cells. As a main inducer of EMT, TGF-β enhances EMT through other signaling pathways such as Wnt/β-catenin, NF-κB, and RTKs, thereby maintaining the interstitial phenotype of tumor cells.^32^ NF-κB plays an important role in EMT,^33–35^ and many carcinogenic molecules promote EMT through the NF-κB pathway, thereby resulting in tumor metastasis.^36,37^ Ren et al. demonstrated that miR-210-3p promotes EMT through sustained activation of NF-κB signaling.^38^ In Fanconi anemia, ROMO1 induces NF-κB-driven EMT by regulating redox status.^39^ In an online database, we found that FERMT1 and NF-κB are positively correlated. Therefore, we wondered whether FERMT1 might promote EMT through NF-κB. We validated this hypothesis through western blotting.

IκBα is an important intermediate molecule that activates the NF-κB signaling pathway.^40^ When degraded, it activates the NF-κB signaling pathway and promotes transcription of target genes ^[43]^. Our results showed that the protein of IκBα was decreased in FERMT1 GC cells and increased in FERMT1 overexpression cells, thus providing evidence that FERMT1 accelerates the degradation of IκBα. The results from Figure 6 further indicated that FERMT1 is a crucial regulator of NF-κB.

In conclusion, this study illustrates FERMT1 plays an important role in GC. We demonstrated that FERMT1 is aberrantly expressed in GC tissues. The expression of FERMT1 is correlated with clinical prognosis in patients with GC. In addition, cell and animal experiments demonstrated that FERMT1 promotes the proliferation, migration, invasion, metastasis, and EMT of GC cells by activating the NF-κB signaling pathway. Therefore, our findings demonstrate that FERMT1 is a new oncogenic factor and they might shed new light on human GC therapy.

## Supplementary Material

Supplemental Material
