# Supplementary material for: FERMT1 promotes gastric cancer progression by activating the NF-κB pathway and predicts poor prognosis
Source: Cancer Biol Ther. 2020 Jul 29;21(9):815–25. doi: 10.1080/15384047.2020.1792218 (PMC7515530; doi:10.1080/15384047.2020.1792218)
Supplement: Supplemental Material [file KCBT_A_1792218_SM3144.docx]

| **Sup.Tab.1 Sequence of primers for Quantitative reverse transcription-PCR** | | |
| --- | --- | --- |
| Gene | Forward primer (5’------3’) | Reverse primer(5’------3’) |
| FERMT1 | AAGCGTCCCTCTTATCGCTG | CTCAAGGCAGTCTCAAGGCA |
| E-cadherin | AGGCCAAGCAGCAGTACATT | CATTCACATCCAGCACATCC |
| N-cadherin | CACTGCTCAGGACCCAGAT | TAAGCCGAGTGATGGTCC |
| Vimentin | CGAAACTTCTCAGCATCACG | GCAGAAAGGCACTTGAAAGC |
| N-cadherin | TTTGAGGGCACATGCAGTAG | ACTGTCCCATTCCAAACCTG |
| MMP9 | CCCCACTTACTTTG GAAACGC | ACCCACGACGATACGATGCTG |
| MMP2 | AGATGCCTGGAATGCCAT | GGTTCTCCAGCTTCAGGTAAT |
| CXCR4 | TCAGTGGCTGACCTCCTCTT | CTTGGCCTTTGACTGTTGGT |
| ICAM1 | ATGCCCAGACATCTGTGTCC | GGGGTCTCTATGCCCAACAA |
| GAPDH | AGAAGGCTGGGGCTCATTTG | AGGGGCCATCCACAGTCTTC |
